# Circulation of Different Lineages of Dengue Virus Type 2 in Central America, Their Evolutionary Time-Scale and Selection Pressure Analysis

**DOI:** 10.1371/journal.pone.0027459

**Published:** 2011-11-04

**Authors:** Germán Añez, Maria E. Morales-Betoulle, Maria Rios

**Affiliations:** 1 Laboratory of Emerging Pathogens, Division of Emerging and Transfusion Transmitted Diseases, Center for Biologics Evaluation and Research, United States Food and Drug Administration, Bethesda, Maryland, United States of America; 2 United States Naval Medical Research Unit No. 3 (NAMRU-3), Cairo, Egypt; Blood Systems Research Institute, United States of America

## Abstract

Dengue is caused by any of the four serotypes of dengue virus (DENV-1 to 4). Each serotype is genetically distant from the others, and each has been subdivided into different genotypes based on phylogenetic analysis. The study of dengue evolution in endemic regions is important since the diagnosis is often made by nucleic acid amplification tests, which depends upon recognition of the viral genome target, and natural occurring mutations can affect the performance of these assays. Here we report for the first time a detailed study of the phylogenetic relationships of DENV-2 from Central America, and report the first fully sequenced DENV-2 strain from Guatemala. Our analysis of the envelope (E) protein and of the open reading frame of strains from Central American countries, between 1999 and 2009, revealed that at least two lineages of the American/Asian genotype of DENV-2 have recently circulated in that region. In occasions the co-circulation of these lineages may have occurred and that has been suggested to play a role in the observed increased severity of clinical cases. Our time-scale analysis indicated that the most recent common ancestor for Central American DENV-2 of the American/Asian genotype existed about 19 years ago. Finally, we report positive selection in DENV-2 from Central America in codons of the genes encoding for C, E, NS2A, NS3, and NS5 proteins. Some of these identified codons are novel findings, described for the first time for any of the DENV-2 genotypes.

## Introduction

Dengue, the most common arboviral disease in the world, is caused by any of the four serotypes of dengue virus (DENV-1 to 4), genus *Flavivirus*, family *Flaviviridae*. DENV is transmitted in its urban cycle through the bite of mosquitoes from the genus *Aedes*, mainly *Aedes aegypti.* DENV RNA encodes 3 structural (Capsid, C; pre-Membrane/Membrane, prM/M and envelope, E) and 7 non-structural proteins (NS1, NS2A, NS2B, NS3, NS4A, NS4B and NS5) [1]. The four DENV are genetically distant from each other and form a number of subtypes (or genotypes) that vary from serotype to serotype [2]. For DENV-2, there are five epidemic genotypes: Asian I (AS-I), Asian II (AS-II), American/Asian (AM/AS), Cosmopolitan (COS) and American (AM), and a sylvatic genotype restricted to strains collected from forests in Africa and South East Asia [3], [4], [5].

DENV was first isolated in the Americas in 1953 and the disease has been present in Central America since 1978, when epidemics caused by DENV-1 were reported in Belize, Guatemala, El Salvador and Honduras [6]. Central America is the isthmian portion of the American continent constituted by Belize, Costa Rica, Guatemala, Honduras, El Salvador, Nicaragua and Panama. That region is epidemiologically dynamic for dengue, where transmission of the four DENV serotypes occurs. It has been suggested that this dynamic involves an active cycle of exchange of viral strains between South and Central America and the Caribbean [2], [7], [8]. Dengue is hyperendemic in Central America as well as in most of the countries of the continent, especially after the re-introduction of DENV-3 in the 90s [9]. Between 1999 and 2010, more than 700,000 cases of dengue have been reported in Central America, >24,000 of which correspond to severe dengue (formerly, dengue hemorrhagic fever) with 468 fatalities, according to data submitted by the countries to the Pan American Health Organization [10] (Figures S1, S2, and S3).

A plethora of phylogenetic studies focused on the evolution of DENV-2 in the Americas have been reported [2], [7], [8], [11], [12], [13], [14]; however, to date no studies have been conducted to analyze the evolutionary aspects of DENV-2 in Central America during the last decade. In this paper, we describe the first fully sequenced DENV-2 from Guatemala, and report the first phylogenetic analyses performed on all available sequences of strains of DENV-2 from countries of Central America, using Maximum likelihood and Bayesian approaches. Our phylogenetic analyses demonstrate that in recent years the circulation of two different lineages of DENV-2 of the AM/AS genotype occurred in the countries of the region. We also discuss, for the first time, aspects on the evolutionary time-scale and selection pressure analysis of circulating DENV-2 in Central America during a period of high dengue activity in the region (1999-2009).

## Methods

### Ethics statement

In this study we used an unlinked leftover specimen that had been collected for laboratory diagnosis, which was made available to us by Dr. Morales-Betoulle for further virological studies. That was considered exempt from written approval by the Institutional Review Board of the FDA (Human Subjects Research - Exempt RIHSC Protocol #127B).

### DENV isolation and detection

For viral detection, we extracted RNA from plasma of a Guatemalan patient with symptomatology of dengue. An aliquot of plasma was also inoculated into C6/36 cells to attempt viral isolation. Viral RNA from plasma or cell culture supernatants was extracted using the QIAamp Viral RNA kit (Qiagen, Gaithersburg, MD) and a DENV serotype-specific quantitative RT-PCR assay (TaqMan) was performed with the RNA-to-Ct 1-step kit in a 7300 Real-Time PCR System (Applied Biosystems, Foster City, CA), following the protocol of Johnson et al. [15]. Results are expressed as PCR detectable units (PDU)/mL.

### Sequencing and phylogenetic analysis of isolated DENV

The entire viral genome of the second passage in C6/36 cells of the isolate termed GU/FDA-GUA09/2009 was amplified by PCR using the One-Step RT-PCR kit (Qiagen) and DENV specific forward and reverse primers, generating 11 overlapping fragments that covered the full genome. The PCR products were purified with the QIAquick PCR purification kit (Qiagen) and sequenced using the BigDye terminator chemistry version 3.1 (Applied Biosystems). The sequences of the 5′ and 3′ untranslated regions of DENV-2 were determined following viral RNA ligation and PCR amplification essentially as described elsewhere [16]. Sequencing reactions were performed on a 3730 DNA analyzer (Applied Biosystems), and the sequences of overlapping fragments were assembled, evaluated and annotated using the software Sequencher, version 4.8 (GeneCodes Corp., Ann Arbor, MI). The full genome sequence of strain GU/FDA-GUA09/2009 was submitted to the GenBank under accession no. HQ999999.

To study the phylogenetic relationships of circulating DENV-2 from Central America, all available sequences from countries of that region were retrieved from the GenBank database. Two datasets were prepared and trees were constructed with the sequences of the E protein gene (1,485 nt), *n = *119; and the complete open reading frame (ORF) of the virus (10,173 nt), *n = *158; through Maximum-likelihood (ML) and Bayesian approaches. Sylvatic strains were used as outgroup to root the trees. The selected Central American strains and additional representative strains collected worldwide from 1944 to 2009, belonging to each of the six DENV-2 genotypes were included and aligned using ClustalX (Table S1). Strains used in this study are presented in the following format: two letters ISO country code/strain name/year of isolation.

The ML analysis was conducted in MEGA5 [17] and the best substitution model obtained from that program was compared with that obtained with the program jModelTest [18]. Bayesian information criterion (BIC) was used to determine the model of nucleotide substitution that best fit the data for each of the subsets analyzed. For the E protein gene analysis, the best fit model was the Tamura-Nei model+Γ (TN93+Γ) and for the ORF, the General Time Reversible (GTR) substitution model with 4 categories of Γ distributed rates plus invariable rates in the rest of the sites (GTR+Γ_4+_I). A bootstrap of 1,000 replicates was used as test of phylogeny.

For each dataset, an additional phylogenetic analysis was undertaken using the Bayesian inference method implemented in the program MrBayes v3.1.2 [19]. For the substitution model, the GTR+Γ_4+_I model with successive branch swapping was used. Five Markov Chain Monte Carlo (MCMC) chains were run for 10,000,000 generations, sampling every 100 generations, with the first 10,000 sampled trees discarded as burnin. Finally, a 50% majority rule consensus tree was constructed from the posterior distribution of trees.

### Natural selection analysis

To assess the selection pressures acting on the codons of the ORF and E protein gene regions of the analyzed DENV-2 strains from Central America, four datasets were prepared, two for the ORF (n = 67 and 143) and two for the E protein gene (n = 24 and 75) (Table S1). We used the HyPhy package under the Datamonkey web-server (www.datamonkey.org) [20]. The ω ratios (*dN/dS*) were calculated using five different likelihood approaches for each of the four datasets: the single-likelihood ancestor (SLAC), Fixed-effects likelihood (FEL), internal branch Fixed-effects likelihood (IFEL), random-effects method (REL), and the partitioning approach for robust inference of selection (PARRIS). For all the methods, GTR model was used as nucleotide substitution bias model, trees were inferred by the neighbor-joining method and significance levels set to p<0.1 or Bayes factor >50.

### Molecular dating analysis

To determine the Time to the Most Recent Common Ancestor (TMRCA) for the analyzed sequences of DENV-2, we used the same dataset of E protein gene sequences employed for the phylogenetic analysis without including the sylvatic strains (*n* = 115) (Table S1). The rate of nucleotide substitution and TMRCA for DENV-2 strains of Central American origin, as well as for all other genotypes, were calculated by using the Bayesian MCMC approach employed by BEAST ver. 1.6.1 [21]. The data was analyzed using the GTR+Γ_4_+I model, using both strict and relaxed uncorrelated lognormal molecular clocks. Bayesian coalescent prior and constant population size tree priors were used to describe the DENV-2 demographic history. Five independent MCMC analyses, each for 10,000,000 steps, were performed for each branch rate model and combined with a burnin value set to 10% generations. The maximum clade credibility tree (MCC) was generated for each model. The 95% highest posterior density (95%HPD) intervals were obtained to ascertain the uncertainty in the parameter estimates.

## Results

### DENV-2 isolation and nucleic acid test results

We detected and isolated DENV from a sample of plasma from a suspected case of dengue from Guatemala. The patient was clinically diagnosed with dengue fever during the outbreak of 2009 and DENV RNA was detected from the blood sample by TaqMan. DENV-2 was identified as the infecting serotype with RNA titers in plasma of 1.29×10^6^ PDU/mL. Plasma was used to infect mosquito C6/36 cells and cytopathic effect, consisting of formation of syncytia and rounding and detachment of the cells from the culture flask was detectable at day 7 and peaked at day 12 post-infection (data not shown). The complete genome of the strain GU/FDA-GUA09/2009 was sequenced, and constitutes the first fully sequenced strain from Guatemala reported to date.

### Phylogenetic analysis of DENV-2 E protein gene and open reading frame

To study the phylogenetic relationships between the strains of DENV-2 circulating in Central America, a search for strains from its constituent countries was conducted in the GenBank database. From Central America, most of the DENV strains deposited in the database were from Nicaragua, from where we retrieved a total of 181 complete genome sequences of DENV-2 spanning from 1999 to 2009. Phylogenetic analyses were performed using the ORF and E protein gene datasets including 141 and 78 Nicaraguan sequences, respectively, after removal of identical sequences. From the remaining countries, only two fully sequenced strains of DENV-2 were available for analysis; one from Belize (BZ/BID-V2952/2002) and our reported strain from Guatemala (Table S1). The E protein gene dataset (n = 119), included selected Nicaraguan strains (n = 78), and sequences from Belize, Costa Rica, El Salvador, Guatemala, Honduras and 2 Mexican strains (MX/12021-Oxkutzcab 01/2001 and MX/13381-Chochola 02/2002) collected from the State of Yucatan [12]. Sequences from Yucatan were included due to the close proximity of this Mexican state to Guatemala. For both datasets (E and ORF) strains from the Caribbean and South America from the 80s and 90s (JM/N.1409/1983, MQ/98-703/1998, CO/PTCOL/1996, and VE/lard3146/1999) were included in the analyses (Table S1).

**Figure 1:**
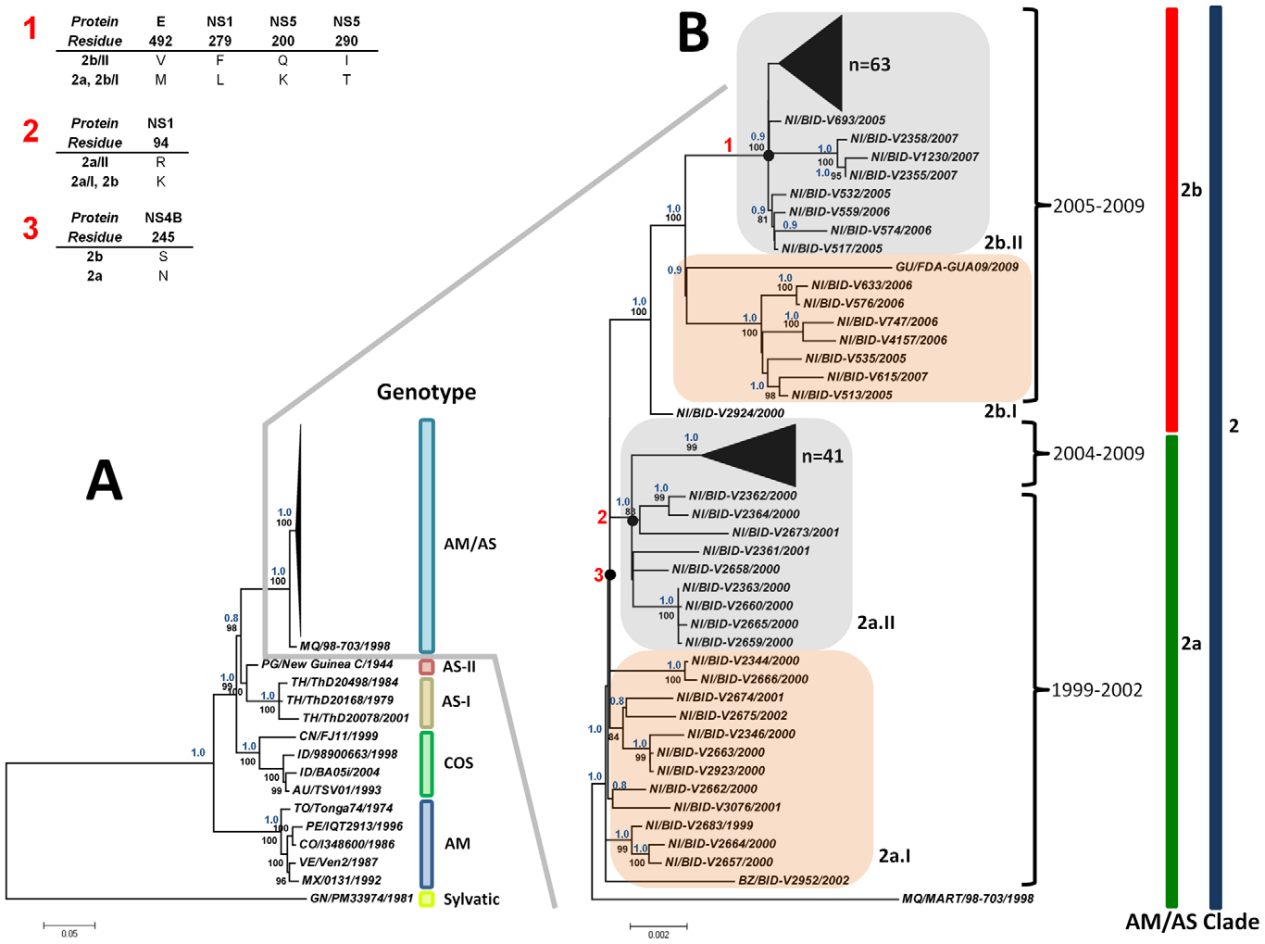
Maximum Likelihood (ML) consensus of the open reading frame (ORF) of DENV-2. A) Consensus ML tree constructed with the ORF of representative sequences of all DENV-2 genotypes. DENV-2 genotypes (Sylvatic, American, Asian I and II, Cosmopolitan and American/Asian) are identified. Strains from the American/Asian genotype are shown collapsed. B) Detail of A showing the cluster of the American/Asian genotype constituted by 141 Nicaraguan strains, and one strain each from Guatemala, Belize and Martinique. Names of each clade and sub-clade are shown, as well as the years of circulation for the sub-clades. Similar sequences at the top of clades 2a and 2b are shown collapsed. Groups I and II within sub-clades 2a and 2b are shown highlighted in a gray and orange shadows. Consensus trees were generated by ML and Bayesian methods. Bootstrap support values of 1,000 replicates using the ML model of evolution and Bayesian posterior probability values are shown in blue above node. Only bootstrapping values >80 are shown. Branch lengths are proportional to the bar and represent the number of nucleotide substitutions between the analyzed viruses. Amino acid changes found in different DENV-2 proteins associated with the different sub-clades are shown and identified in the tree with numbers (1–3) in red.

Analysis of the consensus ML tree built with the nucleotide sequences of the ORF DENV-2 isolates of Central American origin and representative strains from other regions, allows for the segregation of the five known epidemic genotypes of the virus supported by both ML high bootstrap support values and Bayesian posterior probabilities. Sylvatic strain GN/PM33974/1981 was used as an outgroup to root the tree (Figure 1A and 1B). The tree topology obtained with this analysis of the coding region is consistent with what has been reported previously in the literature [22]. Analysis of this tree enabled us to identify two different clades within the strains of the AM/AS genotype that have been circulating in the Americas: clade 1 at the bottom of the sub-tree and constituted in this analysis by older isolates from Jamaica, Colombia and Venezuela, and clade 2, that includes the remaining strains (strain MQ/98-703/1998 from Martinique and all the Central American strains: the analyzed Nicaraguan strains and the strains from Belize BZ/BID-V2952/2002 and Guatemala GU/FDA-GUA09/2009). Clade 2 can be further divided into two sub-clades termed here sub-clades 2a and 2b (Figure 1B).

The phylogenetic tree generated with the ORF sequences also allows for the identification of a temporal-spatial distribution of DENV-2 strains from Nicaragua clustering in both sub-clades 2a and 2b, in which viruses from each of the two sub-clades have co-circulated with members of the other at least since 2005. Unfortunately, there are only a few DENV-2 sequences from 2004 and no sequences from 2003 from Nicaragua available in the database to allow for the understanding of the evolutionary scenario that has driven the appearance of these two lineages of DENV-2 of AM/AS genotype in the region around that time.

Since most sequences from other countries of Central America were from the E protein gene or the three structural genes (i.e. C-prM/M-E), we performed a phylogenetic analysis of E protein gene sequences with these strains. The consensus trees from the phylogenetic analysis obtained by ML and Bayesian methods also show the typical distribution of DENV-2 into its known genotypes with high bootstrapping support values. Similar topology exists between the trees generated with the ORF and E protein gene sequences; i.e. the two sub-clades of the AM/AS genotype identified in the tree generated with ORF sequences were also observed in the tree obtained with sequences of the E protein gene alone.

In the case of the E protein gene, within clade 2a we found clustered strains from Mexico and all countries of Central America included in the analysis (i.e. Belize, El Salvador, Costa Rica, Guatemala and Nicaragua), except for Panama that had no sequence available for study. Within clade 2a, two groups can be identified which are temporarily-associated: at the base of the tree, group 2a.I which includes the oldest DENV-2 sequences from Central America available for this analysis (from Nicaragua strains NI/BID-V2683/1999, NI/BID-V2666/2000 and NI/BID-V2361/2001, strain ES/18 99/1999 from El Salvador, strain BZ/BID-V2952/2002 from Belize and the two Mexican strains from Yucatan included (MX/12021/Oxkutzcab/2001 and MX/13381/Chochola/2002), and a recent strain from Guatemala (GU/F07-030/2007). The second group (2a.II) is constituted exclusively by Nicaraguan strains collected in the period 2004–2009.

**Figure 2:**
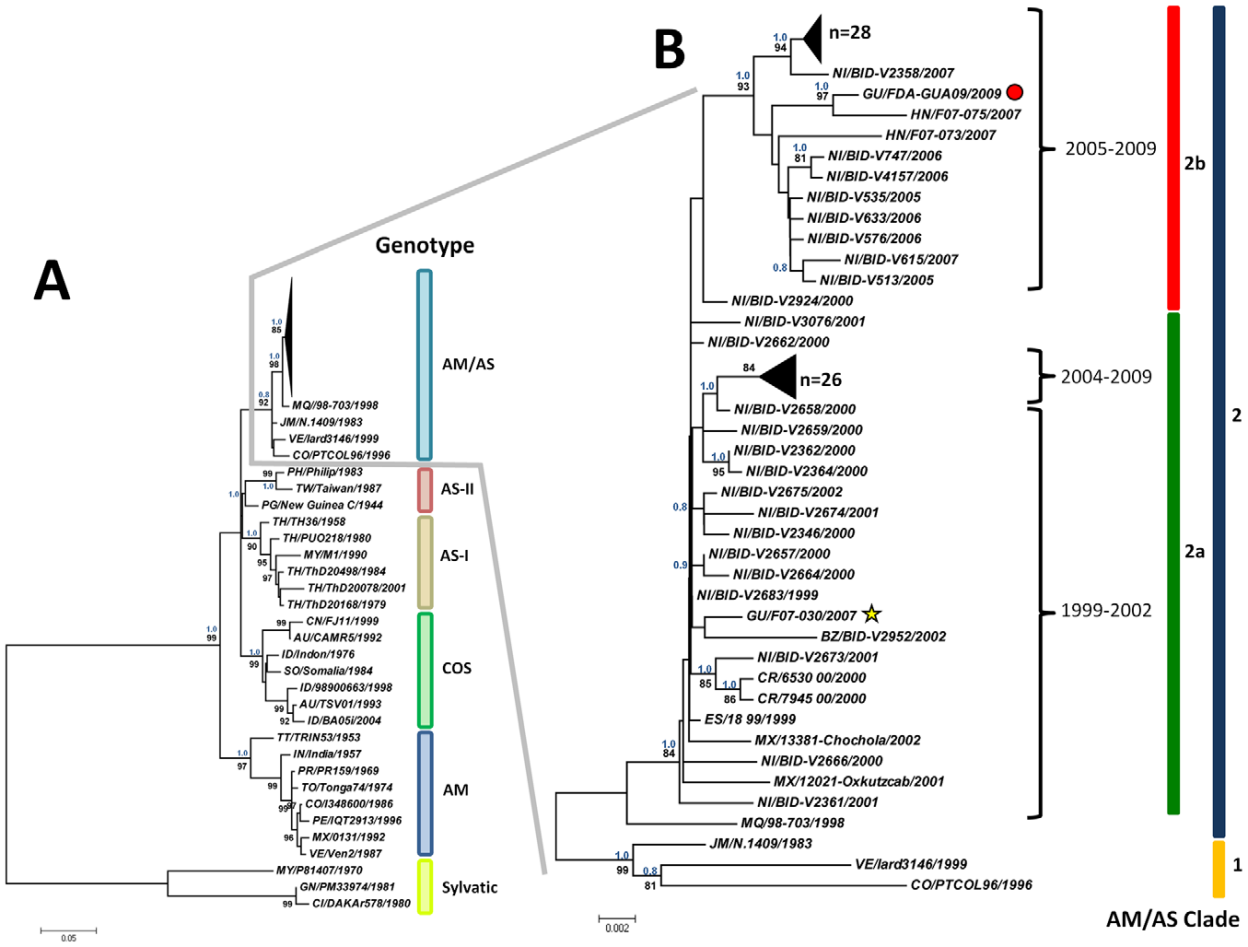
Maximum Likelihood (ML) consensus tree of the Envelope protein gene of DENV-2. A) The phylogenetic relationships between members of all DENV-2 genotypes based on the sequences of the Envelope protein gene. DENV-2 genotypes (Sylvatic, American, Asian I and II, Cosmopolitan and American/Asian) are identified. B) ML tree showing in details the relationships between Central American and Mexican isolates collected between 1999 and 2009. Similar sequences at the top of clades 2a and 2b are shown collapsed. A red circle and a yellow star denote strains GU/FDA-GUA09/2009 and GU/F07-030/2007, respectively. Consensus trees were generated by ML and Bayesian methods. Bootstrap support values of 1,000 replicates using the ML model of evolution and Bayesian posterior probability values are shown above and below each node. Only bootstrapping values >80 are shown. Branch lengths are proportional to the bar and represent the number of nucleotide substitutions between the analyzed viruses.

The other sub-clade (clade 2b) also forms two groups: 2b.I, formed by recent strains from Nicaragua, Honduras and the strain here described as GU/FDA-GUA09/2009 from Guatemala, and group 2b.II, constituted exclusively by Nicaraguan strains (Figure 1B). Strain NI/BID-V2924/2000 is located at the base of clade 2b suggesting its possible role as a representative of the origin of this clade (Figures 1B and 2B). As can be seen in Figure 2, the two Guatemalan strains in the analysis (GU/F07-030/2007 and GU/FDA-GUA09/2009) clustered in different clades (2a and 2b, respectively), suggesting that these two lineages of DENV-2 have been circulating in recent times in Guatemala.

On the other hand, Nicaraguan strains are temporal and spatially spread between clades 2a and 2b. Strains collected prior to 2005 tend to cluster in clade 2a, while strains collected after 2005 cluster in either sub-clade 2a and 2b. That observation suggests that different lineages of DENV-2 have circulated in Nicaragua in the recent past, and both clades have co-circulated there during the last decade epidemics (Figure 2B). Due to the paucity of availability of DENV-2 strains from other countries of Central America it is hard to speculate whether the evolutionary scenario of DENV-2 in those countries have been similar to that observed for Guatemala and Nicaragua.

The Caribbean is believed to play an important role in the circulation of DENV-2 strains, with importations and exportations occurring from and to Central and South America [2], [7], [8], [11]. To further help in understanding DENV-2 evolutionary dynamics through the region, we performed a phylogenetic analysis of the ORF of 170 DENV-2 strains (Table S2) using both ML and Bayesian approaches. This analysis, included the same dataset of Central American strains used in our analysis of the ORF plus strains from the Caribbean and South America that were reported by McElroy et al [7]. Analysis of this tree revealed that clade 1 of our classification included most of the strains from Puerto Rico (13/16), along with the Brazilian strain BR64022 from 1998, and two strains from the US Virgin Islands (strains BID-V2946 and BID-V2948, from 1987 and 1990, respectively). The clade 2 of our classification included Puerto Rican strains BID-V1032/1998, BID-V600/2005 and BID-V595/2006, strain BID-V2960/2005 from the US Virgin Islands, and the strains from Dominican Republic, Saint Kitts and Nevis, and Cuba. Those are closely related to strain MQ/98-703/1998 from Martinique, and distant to the DENV-2 strains from Central America (Figure S4), altogether suggesting that in situ evolution had happened in Central America and the Caribbean after introduction of the AM/AS genotype in that region.

### Selection analysis for E and the complete coding regions of DENV-2

Dengue epidemic transmission cycle is complex, and involves two different types of host: the invertebrate vector (*Aedes sp.*) and the human host. Under the pressure applied by these two different environments, strains of DENV with higher fitness to successfully adapt to these challenges are subjected to natural selection [23]. To study the selection pressure exerted over DENV-2 in Central America, two datasets for the E protein gene (n = 24 and n = 75) and two for the ORF (n = 67 and n = 143) (Table 1), where analyzed by the FEL, IFEL, REL, SLAC and PARRIS methods. In the case of the FEL, IFEL, SLAC and PARRIS codons with p values ≤0.1 were considered under positive selection, and for REL a Bayes factor ≥50 was considered positive.

**Table 1:** Selection pressure analysis of the open reading frame (ORF) (3391 codons) and E protein gene (495 codons) datasets of DENV-2 from Central America, using the FEL, IFEL and SLAC methods employed in the Datamonkey server (www.datamonkey.org).

Dataset	Codon	Protein and amino acid residue	FEL		IFEL		SLAC	
			ω	*P* value	ω	*P* value	ω	*P* value
E (n = 24)								
	*131*	*E_131_*	*36.11*	*0.22*	*149.33*	*0.06*	0.25	0.53
E (n = 75)								
	*83*	*E_83_*	*25.35*	*0.11*	*49.05*	*0.08*	0.52	0.50
ORF (n = 143)								
	1129	NS2A_2_ *	28.89	0.06	−0.00	1	1.90	0.34
	*2760*	*NS5_269_* *	*34.91*	*0.05*	*34.23*	*0.11*	1.90	0.34
	**3014**	**NS5_523_**	**44.70**	**0.03**	**39.94**	**0.09**	1.98	0.30
	*3341*	*NS5_850_*	*10.28*	*0.23*	*30.81*	*0.09*	1.17	0.39
ORF (n = 67)								
	*41*	*C_41_* **	*15.79*	*0.29*	*61.71*	*0.09*	0.07	0.67
	*363*	*E_83_* **	*19.92*	*0.24*	*77.77*	*0.07*	0.07	0.69
	409	E_129_ **	37.27	0.09	−0.00	1	0.22	0.30
	*1655*	*NS3_180_* **	*20.04*	*0.24*	*78.17*	*0.07*	0.07	0.70
	*1912*	*NS3_437_* **	*20.90*	*0.24*	*81.46*	*0.07*	0.07	0.67
	**3014**	**NS5_523_**	**64.46**	**0.04**	**98.23**	**0.06**	0.22	0.30
	*3341*	*NS5_850_*	*16.49*	*0.23*	*65.91*	*0.06*	0.13	0.37

Sites found under positive selection by two methods are shown in bold, while sites found by only one method but with other method close to the significance threshold value are shown in italics.

*Site only detected in the ORF dataset, n = 143.

**Site only detected in the ORF dataset, n = 67.

Low ω ratios (0.05–0.09) obtained from these approaches suggest that codons in DENV-2 from AM/AS genotype from Central American origin are under strong negative selection, as previously pointed for this and other genotypes of DENV-2 [24], [25], [26]. Our analysis of selection in ORF sequences of DENV-2 of the AM/AS genotype has involved the higher number of analyzed sequences in comparison with all the previous analyses published on the subject to date [27]. In general terms, we found that the ability of the employed approaches to identify codons under positive selection will greatly depend on the number of sequences available for the analysis. Differences in the size of datasets studied for E and ORF sequences of DENV-2 of AM/AS genotype rendered different sites subjected to positive pressure.

In the two E protein gene datasets, two codons were found to be under weak positive pressure, only by the IFEL method: E_131_ in the n = 24 dataset, ω = 149.33, p = 0.06 and E_83_ in the n = 75 dataset, ω = 49.05, p = 0.08. Codon E_83_ had a FEL p value of 0.11, which is close to the established significant threshold of 0.1 (Table 1). No codon under positive pressure was detected by the REL, SLAC and PARRIS methods for these two datasets using the defined p value and Bayes factor threshold.

In the two ORF datasets analyzed, we were able to find codons under positive pressure depending on the number of strains analyzed in each dataset. In the dataset of 67 strains, we found seven codons under weak positive pressure in the C (codon 41, IFEL ω = 61.71, p = 0.09), E (codon 83, IFEL ω = 77.77, p = 0.07, and codon 129, FEL ω = 37.27, p = 0.09), NS3 (codon 180, IFEL ω = 78.17, p = 0.07, and codon 437, ω = 81.46, p = 0.07) and NS5 proteins (codon 523, detected by both FEL and IFEL methods ω = 64.46 and 98.23, p = 0.04 and 0.06, respectively). All the codons detected by IFEL had FEL p values close to the threshold of 0.1 (Table 1). As with the E protein gene datasets, REL and PARRIS failed to find codons under positive pressure in the ORF datasets.

Four codons under weak positive pressure were found in the dataset of 143 strains, one in the NS2A (codon 2, FEL ω = 28.89, p = 0.06) and three in NS5 gene (codon 269, FEL ω = 34.91, p = 0.05, codon 523, FEL ω = 44.70, p = 0.03 and IFEL ω = 39.94, p = 0.09; and codon 850, IFEL ω = 30.81, p = 0.09. As for the 67 strains dataset, the only codon detected by both the FEL and IFEL methods was NS5_523_. REL and PARRIS analyses were not able to be conducted on the 143 strains dataset due to the limit of sequences allowed by the Datamonkey server. For both ORF datasets (i.e. n = 67 and n = 143), no codon under positive pressure was detected by the SLAC method, when using the pre-established significance threshold at p<0.1. Results of the SLAC method are shown for the sites detected by FEL and/or IFEL methods for the two ORF datasets (Table 1).

### Molecular clock analysis

In order to better understand the epidemiological scenario responsible for the replacement process that DENV-2 underwent in Central America (i.e. substitution of the autochthonous AM genotype with the emergence and endurance of the AM/AS genotype), we performed a time-scale analysis to know the Time to the Most Recent Common Ancestor (TMRCA) for the AM/AS genotype of DENV-2 that has been circulating in Central America in the recent past. The results of the molecular clock analysis of E gene sequences of DENV-2 are shown in Table 2. An extended version of this Table including the Effective Sample Size (ESS) for the studied parameters can be found in Table S3.

**Table 2:** Molecular clock analysis of DENV-2 from all genotypes used in the study without including sylvatic strains.

Model	Prior (mean)	Posterior (mean)	Marginal likelihood (mean±stderr)	Substitution rate mean (10^–4^ substitutions/site/year [95%HPD])	TMRCA (mean no. of years [95%HPD])			
Bayesian coalescent pior:					All Genotypes (root age)	AM/AS genotype	AM/AS genotype, clade 2	AM/AS genotype, clade 2b
**GTR+**Γ**+I, relaxed lognormal clock**	**−538.11**	**−8018.52**	**−7480.41±0.34**	**7.46 (6.32–8.68)**	**121 (105–138)**	**32 (27–36)**	**19 (15–22)**	**11 (9–12)**
GTR+Γ+I, strict clock	−548.27	−8030.81	−7482.53±0.35	7.58 (6.43–8.79)	119 (104–135)	31 (27–35)	18 (15–22)	11 (9–13)
Constant size:								
GTR+Γ+I, relaxed lognormal clock	−538.73	−8022.66	−7483.93±0.33	7.49 (6.38–8.63)	120 (106–135)	32 (28–36)	18 (15–22)	11 (9–12)
GTR+Γ+I, strict clock	−549.27	−8035.01	−7485.73±0.24	7.59 (6.48–8.80)	118 (105–133)	31 (27–34)	18 (15–22)	11 (9–13)

Best-fit model is shown in bold. GTR+Γ_4_+I, General Time Reversible (GTR) substitution model with 4 categories of Γ plus invariable rates; 95% HPD, 95% highest probability density; TMRCA, time to the most recent common ancestor; AM/AS, American/Asian.

**Figure 3:**
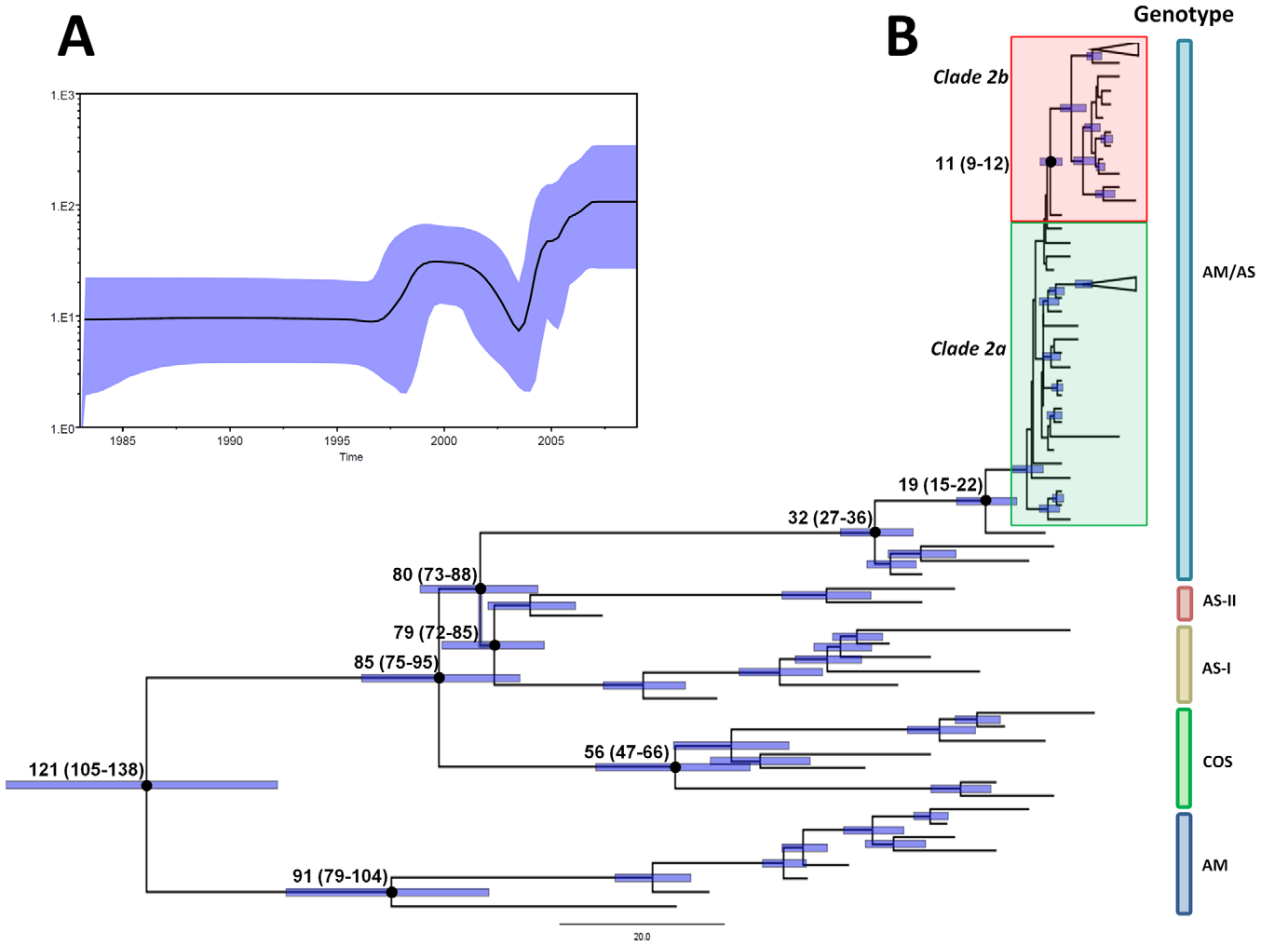
Maximum clade credibility (MCC) tree and Bayesian Skyline plot of the analyzed DENV-2. A) Bayesian coalescent inference of genetic diversity and population dynamics using the Bayesian Skyline plot available in BEAST 1.6.1., for DENV-2 strains of American/Asian genotype in Central America. X axis represents years of study and y axis the relative genetic diversity product of the effective population size. Black line represents the mean estimate and the blue shadow, the 95%HPD. B) Maximum clade credibility tree derived from the Bayesian analysis of the envelope protein of DENV-2 with the best fit model (relaxed lognormal clock), showing the mean time to the most recent common ancestor (TMRCA) in each principal node (mean and 95%HPD). The 95% highest probability density (95%HPD) for each node age, are shown as blue bars. DENV-2 genotypes (Sylvatic, American, Asian I and II, Cosmopolitan and American/Asian) are identified. Clades 2a and 2b are highlighted in a green and red shadow, respectively.

The results revealed that the best fitted model was the relaxed lognormal clock model and Bayesian coalescent prior analysis. Under this model, the mean substitution rate was about 7.46×10^–4^ substitutions/site/year, with 95%HPD of 6.32–8.68×10^–4^ substitutions/site/year. The MCC tree generated under the best fit model and the Bayesian Skyline (BS) plot analysis for Central American DENV-2 strains of AM/AS genotype are shown in Figure 3. The TMRCA for all genotypes (excluding sylvatic strains, *n* = 115) was between 105 and 138 years, mean of 121 years, which is congruent with previous findings [10]. In the case of the AM/AS genotype, the TMRCA appears to have only existed recently, around 32 years ago (95% HPD: 27–36). Between clades 1 and 2 of the AM/AS genotype, the TMRCA was about 19 years ago (95% HPD: 15–22), while for clade 2a and 2b, the TMRCA existed about 11 years ago (95% HPD: 9–12) (Table 2).

The BS analysis shows a sharp increase in the diversity that started around 1997–1998, coinciding with the large epidemic caused by DENV-1 and DENV-2 in Nicaragua in 1998 [10], [28], after a period of genetic stability from 1983 to 1997. This diversity reached a plateau that remained steady until the end of 2001, when the genetic diversity of the virus came down quickly to the same levels observed before 1996. That low diversity was maintained until 2003. Around the end of 2003 and the beginning of 2004 another sharp increase in diversity occurred reaching higher levels of genetic variability than those observed before, peaking around 2006. That trend was sustained until 2009, the last year sampled in our study. The observed high genetic diversity in circulating DENV-2 in Central American together with the results from our phylogenetic analysis, suggest that the virus is undergoing evolution in situ. That together with the geographical expansion of emerging clades of the virus may result in an increased number of cases of severe dengue in the countries of the region.

## Discussion

We analyzed the phylogenetic relationships of DENV-2 strains isolated from countries of Central America from 1999 to 2009. To the best of our knowledge, this is the first report on the phylogeny, molecular clock and selection pressure analysis of DENV-2 in this region. We also report here the first complete genome sequence of a Guatemalan DENV strain, and the second DENV-2 ever sequenced from that country. Our phylogenetic analysis suggests that two lineages of DENV-2 of AM/AS genotype have circulated in Central America and in Nicaragua, they have co-circulated at a certain point in the recent past.

An inherent limitation of this analysis is the fact that most sequences of DENV-2 strains from Central America available in the GenBank are from Nicaragua. Only a very small number of sequences available are from the rest of the countries of Central America, and those are mostly partial sequences. Thus, additional fully sequenced strains of DENV from other countries of Central America are needed in order to perform analyses to better understand the intricacies on the dynamics of the circulation of DENV in that region.

The phylogeny of DENV-2 in Central America has been largely hypothesized to be reflected by the Nicaraguan scenario, from where an important number of sequences spanning more than a decade of collections are available for study. The exact year in which the AM/AS genotype of DENV-2 was introduced into countries of Central America other than Nicaragua, and its complete phylogenetic history is hard to trace due to the unavailability of sequences spanning the last 15 years, time in which that genotype started to replace all other genotypes in the continent [2]. Data from our molecular clock analysis suggest the TMRCA for the AM/AS genotype strains from Central America existed around 19 years ago (95%HPD 15–22 years).

The first reference to the origin of Nicaraguan DENV-2 strains was made during the epidemic of 1998, where Balmaseda et al [28] using restriction site-specific PCR (RSS-PCR) identified the isolates circulating as belonging to the “Jamaica” subtype (AM/AS genotype). More recently within the Pediatric Dengue Cohort Study (PDCS) conducted in that country since 2004, a number of strains have been sequenced and phylogeographic analyses have been conducted identifying two clades of DENV-2 circulating in its capital city Managua. These clades, denominated “clade 1” and “clade 2” by Balmaseda [29], correspond respectively to clades 2a and 2b in this study. One of these circulating clades (“clade 2” in Balmaseda's nomenclature) has been associated with severe dengue and also with the appearance of a higher proportion of symptomatic dengue cases in the cohort during 2007-2008 [29], [30]. In those studies, the authors suggest that the older “clade 1” circulated during 2004–2005 and 2005–2006 and “clade 2” was only found during 2006–2007 and 2007–2008. In our analysis, we have found that a number of strains from 2004 and 2005 (therefore corresponding to their “clade 1”) clustered together with strains from 2007 and 2009 (Figures 1B and 2B). That finding suggest that the co-circulation of both clades still happened in later years, perhaps in lower frequency than before, but in fact the new “clade 2” has not totally replaced “clade 1” in Nicaragua. In our analysis, the TMRCA for clade 2b (that includes strains from Nicaragua, Honduras and Guatemala) appears between 9 and 12 years ago (mean 11 years), coinciding with the occurrence of large DENV epidemics associated with circulation of DENV-2 with any of the other serotypes in the countries of that region [10].

While in Nicaragua strains from this AM/AS genotype has been linked to severe dengue, this has not been the case in other countries of that region [10], [29]. However, the low number of cases of severe dengue reported in Belize, Costa Rica, El Salvador, Guatemala, and Panama most likely result from under-reporting or misdiagnosis, which have been proven to occur in other dengue endemic countries in the Americas [31]. On the other hand, Honduras is by far the country that has reported more cases of dengue, dengue hemorrhagic fever/severe dengue and deaths associated with the disease in Central America in the last decade (Figures S1, S2, and S3). Interestingly, it may have happened that opposed to other Central American countries that may have been underreporting, in Honduras cases of other febrile diseases may have been accounted as dengue cases, which were most likely not confirmed by laboratory tests. Consequently, the number of symptomatic dengue cases in that country may be over reported. This interesting epidemiological question deserves to be studied in more detail.

Another limitation encountered when performing such epidemiological analyses in that the official statistics available from PAHO, which are received directly from the Ministries of Health from the countries of the region, sometimes reflects the number of suspected dengue cases while in others refer to confirmed cases only, as is the case of Nicaragua [10]. Even in the case of Nicaragua, recent reports show significant differences in the number of confirmed symptomatic dengue cases obtained in the PDSC study in comparison with data provided by the Nicaraguan Ministry of Health, which is used as source for the official statistics compiled by the PAHO [32].

Central America represents an interesting place to the evolutionary dynamic of dengue. The region is surrounded by the Caribbean and South America, both of which have been continuous foci of dengue transmission in the last decades [2], [8], [14], [26]. DENV-2 strains from Asian origin have replaced the autochthonous AM strains that were circulating in the region during the 80s and 90s [5]. This has been linked, at least in a part, to the increasing number of severe dengue cases reported in the continent [13], [33].

Circulation of DENV-2 strains from three genotypes and its ultimate replacement by strains of the AM/AS genotype has been reported in Central America. Bennet et al. [2] reports phylogenetic analyses that includes a number of Central American strains of the AS-I genotype (strain ES 12 00 from El Salvador), AM genotype (strains CR A and B from Costa Rica) and the AM/AS genotype (CR 6530/2000 and CR 7945/2000 from Costa Rica and strain ES 18 99/1999 from El Salvador). All these AM/AS genotype sequences were included in our E protein gene phylogenetic analysis. Similarly, Foster et al [8] found four Honduran strains from 1991 (HON A–D) and a single strain from El Salvador (SAL 1987) belonging to the AM genotype, while a single Honduran strain (HON 1986) was identified to be closely related with the prototype strain New Guinea C (NGC), from the AS-II genotype.

Circulation of strains of the AS-I or AS-II genotypes in the Americas has been previously reported in countries outside Central America (Venezuela, Cuba, Haiti and Mexico) [13], [26], [34], [35]. However, that seems to be unlikely and possibly the resultant of laboratory contamination with the strain NGC, a commonly used prototype strain present in most of the laboratories involved in dengue research throughout the world. Cases of NGC-like strains have been detected and reported even in different regions outside the Americas (i.e. China and Vietnam [26]), but none of these NGC-like strains have the amount of variation expected based on the consistently reported substitution rates to which epidemic DENV-2 would be subjected in nature [8], [36]. On the other hand, a single strain isolated in 1996 (strain BC17) from the Mexican state of Yucatan which is a neighbor of Guatemala, has been reported to cluster within the COS genotype [34]. That finding has been considered as evidence of limited circulation of this genotype in that region, although as in the case of the strains of AS-I and AS-II genotypes identified circulating in the continent, it may also be the result of erroneous identity and potential contamination.

Our analysis shows that strains GU/FDA-GUA09/2009 and HN/F07-075/2007 constitute a different lineage within sub-clade 2b of the AM/AS genotype of DENV-2. This finding is supported by high bootstrap values in ML analysis and Bayesian high posterior probability values. Thus, our analysis confirmed that this linage of the AM/AS genotype represented by GU/FDA-GUA09/2009 and HN/F07-075/2007 strains, circulated in 2009 in Guatemala. These findings agree with the suggestion by Tang et al. [37] that the strains currently present in Guatemala and Honduras may be the result of re-emergence of autochthonous DENV-2 strains introduced sometime in the past, instead of being newly introduced viruses. If this is true, it is possible to speculate that there is more than one lineage of DENV-2 circulating in these countries, and that idea can be supported by our results that also identified at least two lineages of DENV-2 circulating simultaneously in neighboring Nicaragua. There is a possibility suggested by the positioning of strain NI/BID-V2924/2000 basal to clade 2b (Figures 1B and 2B) that in the past, strains closely related to NI/BID-V2924/2000 were introduced from Nicaragua to neighboring Guatemala and Honduras, and that continuous introduction resulted in clade replacement by strains with higher fitness, followed by in situ evolution of these lineages. This may be an epidemiological feature of DENV-2 transmission dynamics in Central America.

In relation to the selection pressure exerted over DENV-2, a number of reports [25], [38] and our own results shown that overall the amino acid sequences in DENV-2 are highly conserved, therefore suggesting that the virus is subjected to strong purifying selection. However, in our analysis we were able to detect codons under weak positive selection in the Central American DENV-2 strains analyzed: two when analyzing E protein gene datasets alone (codons 83 and 131) and nine when analyzing ORF datasets (codons C_41_, E_83_ and _129_, NS2A_2_, NS3_180_ and _437_, NS5_269_, _523_ and _850_) (Table 1).

The findings of positive selection in codons of E are expected since this protein is subjected to immune pressure and a number of codons in E have been reported to be subjected to this type of selection. From the three codons found under positive selection in the Central America datasets used in our analysis two (129 and 131) have been reported before by Twiddy et al and Bennet et al [2], [26], [39] when analyzing datasets of Cosmopolitan, Asian and AM/AS DENV-2 strains, but codon 83 identified by our analysis appears to be a novel addition to the already numerous sites identified under positive pressure in the E protein gene to date (reviewed in [27]). In congruence with Vasilakis et al. [25], we did not find evidence of positive selection on E protein position 390, which has been suggested as a virulence marker for DENV-2 [33]. However, we found that the mutation D→N_390_ was fixed in all the analyzed DENV-2 strains from Central America since 1999 (G. Añez, information not published).

We also detected for the first time positive selection in a codon of the capsid of the virus (C_41_), close to the hydrophobic conserved region of the protein [40], although only when using the IFEL method and very close to the statistical significant threshold. The capsid of DENV contains the genome viral, and no site under positive pressure had been reported for any of the DENV serotypes to date. However, it may be possible that in the in situ evolution scenario that the AM/AS genotype of DENV-2 is undergoing in Central America, viruses with amino acids residues responsible for advantageous replication are been selected in this protein, and that the role of the adaptive pressure over this site in the capsid protein needs to be properly addressed by detailed studies.

We are also reporting for the first time all sites detected by our analyses under positive pressure in the NS2A, NS3 and NS5 genes. Since the function of the small NS2A protein is not well understood, it is hard to speculate about the significance of codons found under positive pressure in this protein. NS2A, NS4A and NS4B has been shown to antagonize the interferon response during DENV infection [41], and it would be evolutionary advantageous for the virus to select for strains with strong innate immunity suppression mechanisms. As for the sites detected in NS3, there have not been reports on positive selection in this protein in none of DENV serotypes before. NS3 is important protein for the replication of the virus, since it possesses protease and ATPase/helicase functions necessary for viral replication [42], and as in the case of the capsid protein, this finding needs to be further investigated.

Three codons were identified under positive pressure in the NS5 protein, which is crucial for viral replication for its RNA- dependent RNA polymerase [43]. All these sites were detected only by one of the methods employed, except for one site (NS5_523_) that was detected by both FEL and the IFEL methods in both ORF datasets. In the case of the other two sites reported in the NS5 protein (269 and 850), these sites were detected by either one of the methods (FEL and IFEL) with statistical significance, while p values from the other method were very close to the established significance limits (Table 1). Jointly, these results should be studied in greater detail to elucidate the possible role of these residues in the pathogenesis of the disease.

Nucleic acid amplification technology (NAT) assays has been developed and implemented in the detection of DENV in endemic countries, as well as in places in which the disease is imported and can cause autochthonous transmission, as it happened recently in the US [44]. Even though primers and probes for these assays are designed to target conserved regions of the viral genome, there is always a risk that the assay could fail to detect variants bearing mutations located at the target area. Monitoring of the occurrence of mutations in DENV appears to be a solution that allows for updating diagnostic NAT assays when needed. The use of complete genome sequencing appears to be more suitable than partial sequencing in order to detect variations that could appear all over the DENV genome and could negatively affect the performance of NAT tests.

## Supporting Information

Figure S1 **Number of cases of dengue and severe dengue (previously dengue hemorrhagic fever) and genotypes circulating in countries of Central America from 1999-2010, as reported by the countries to the Pan American Health Organization (PAHO).**
(TIF)

Figure S2 **Number of cases of severe dengue (previously dengue hemorrhagic fever) in countries of Central America from 1999-2010, as reported by the countries to the Pan American Health Organization (PAHO).**
(TIF)

Figure S3 **Number of deaths attributed to dengue in countries of Central America from 1999-2010, as reported by the countries to the Pan American Health Organization (PAHO).**
(TIF)

Figure S4 **Maximum likelihood consensus tree of the open reading frame of DENV-2 strains from Central America, the Caribbean and South America.**
(TIF)

Table S1 List of DENV-2 strains used for phylogenetic, molecular clock and selection pressure analyses, by country and year of isolation.
(DOCX)

Table S2 List of DENV-2 strains of American/Asian genotype used for phylogenetic analysis of ORF sequences between circulating strains of Central American and Caribbean origin (n = 170), by country, year of isolation and clade.
(DOCX)

Table S3 Molecular clock analysis of DENV-2 from all genotypes used in the study, showing the Effective Sample Size (ESS) for the studied parameters.
(DOCX)
