# Supplementary figures and images for: Circulation of Different Lineages of Dengue Virus Type 2 in Central America, Their Evolutionary Time-Scale and Selection Pressure Analysis
Source: PLoS One. 2011 Nov 4;6(11):e27459. doi: 10.1371/journal.pone.0027459 (PMC3208639; doi:10.1371/journal.pone.0027459)

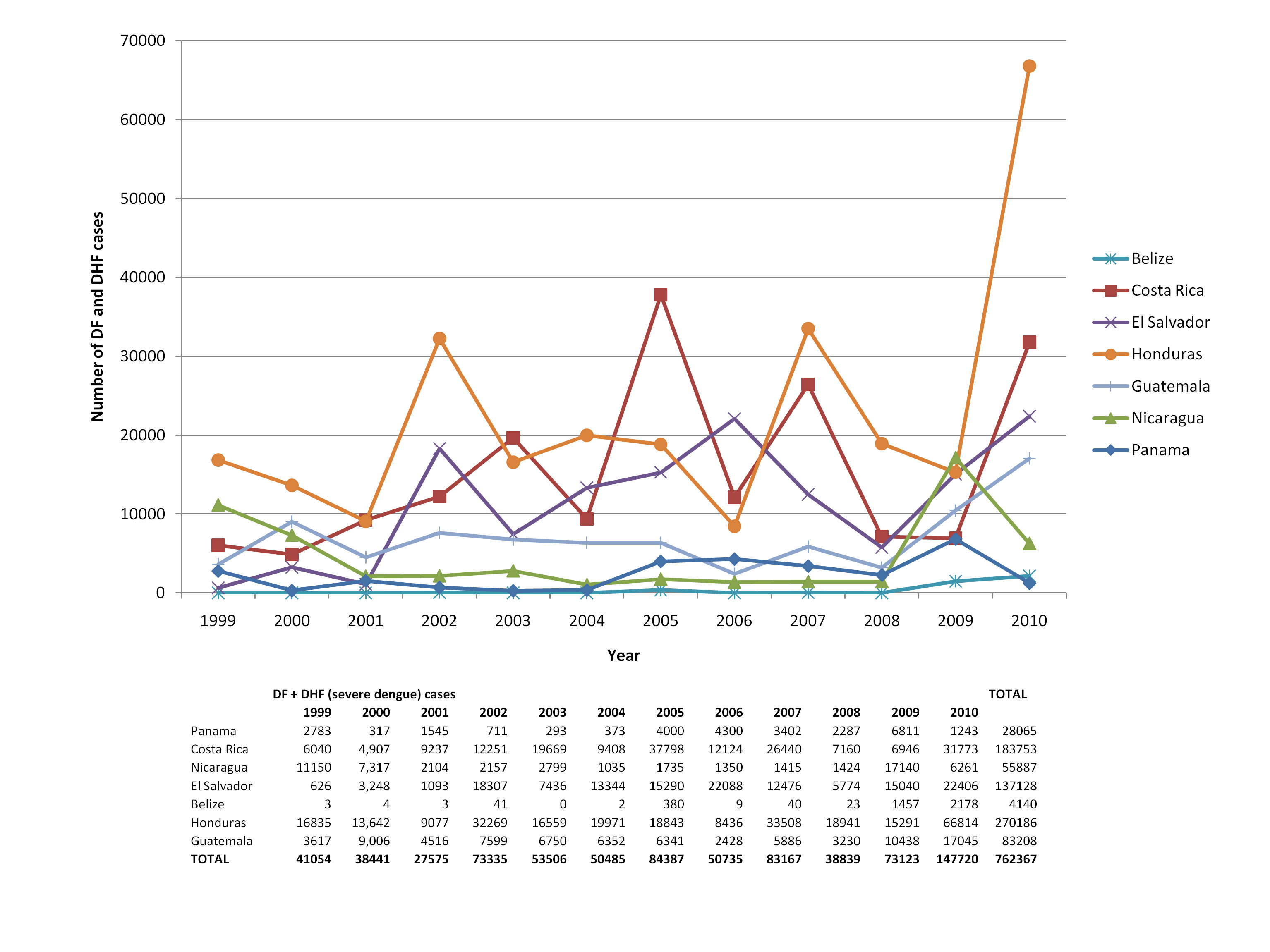

Supplement: Figure S1 — Number of cases of dengue and severe dengue (previously dengue hemorrhagic fever) and genotypes circulating in countries of Central America from 1999-2010, as reported by the countries to the Pan American Health Organization (PAHO). (TIF) [file pone.0027459.s001.tif]

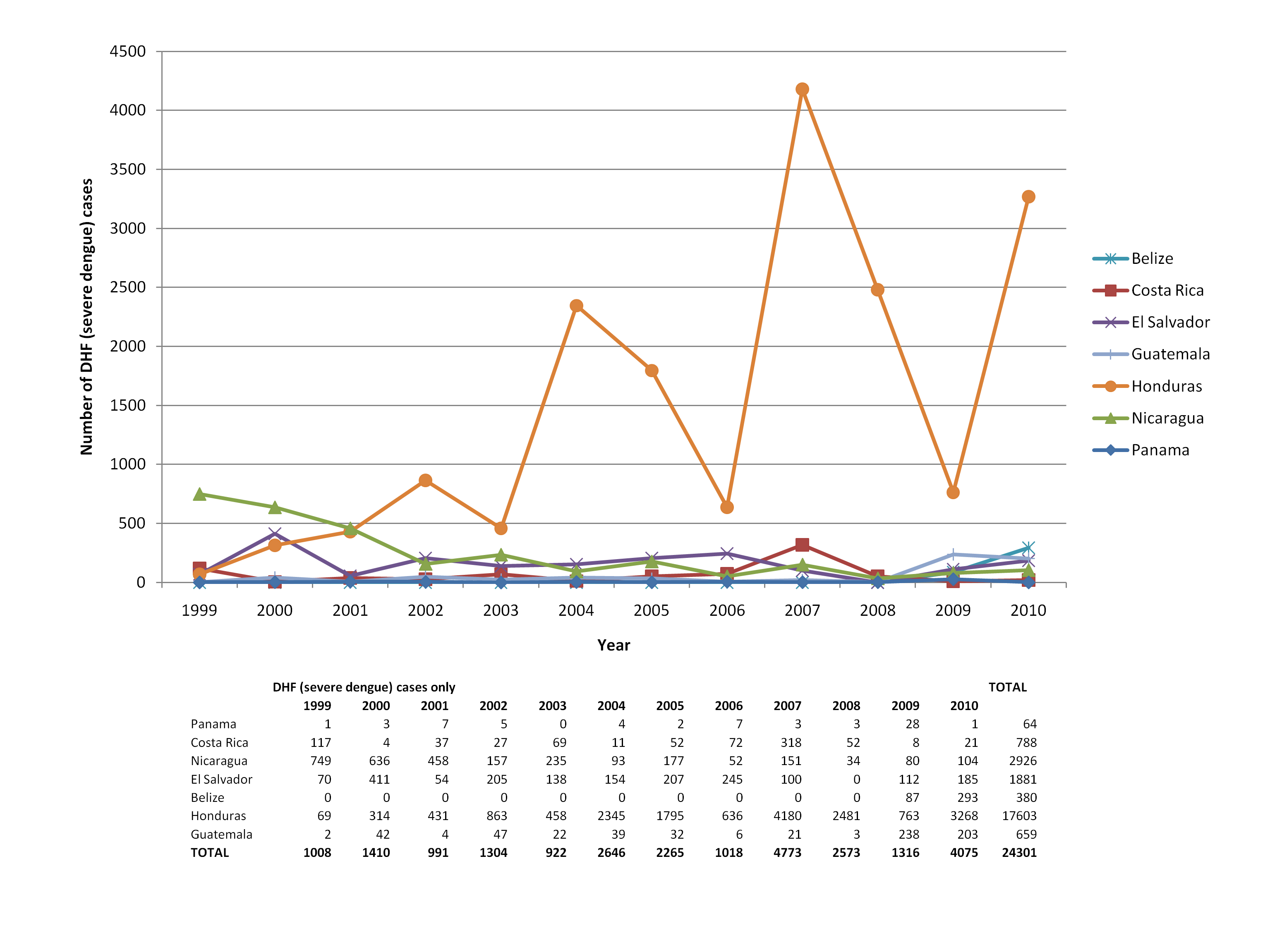

Supplement: Figure S2 — Number of cases of severe dengue (previously dengue hemorrhagic fever) in countries of Central America from 1999-2010, as reported by the countries to the Pan American Health Organization (PAHO). (TIF) [file pone.0027459.s002.tif]

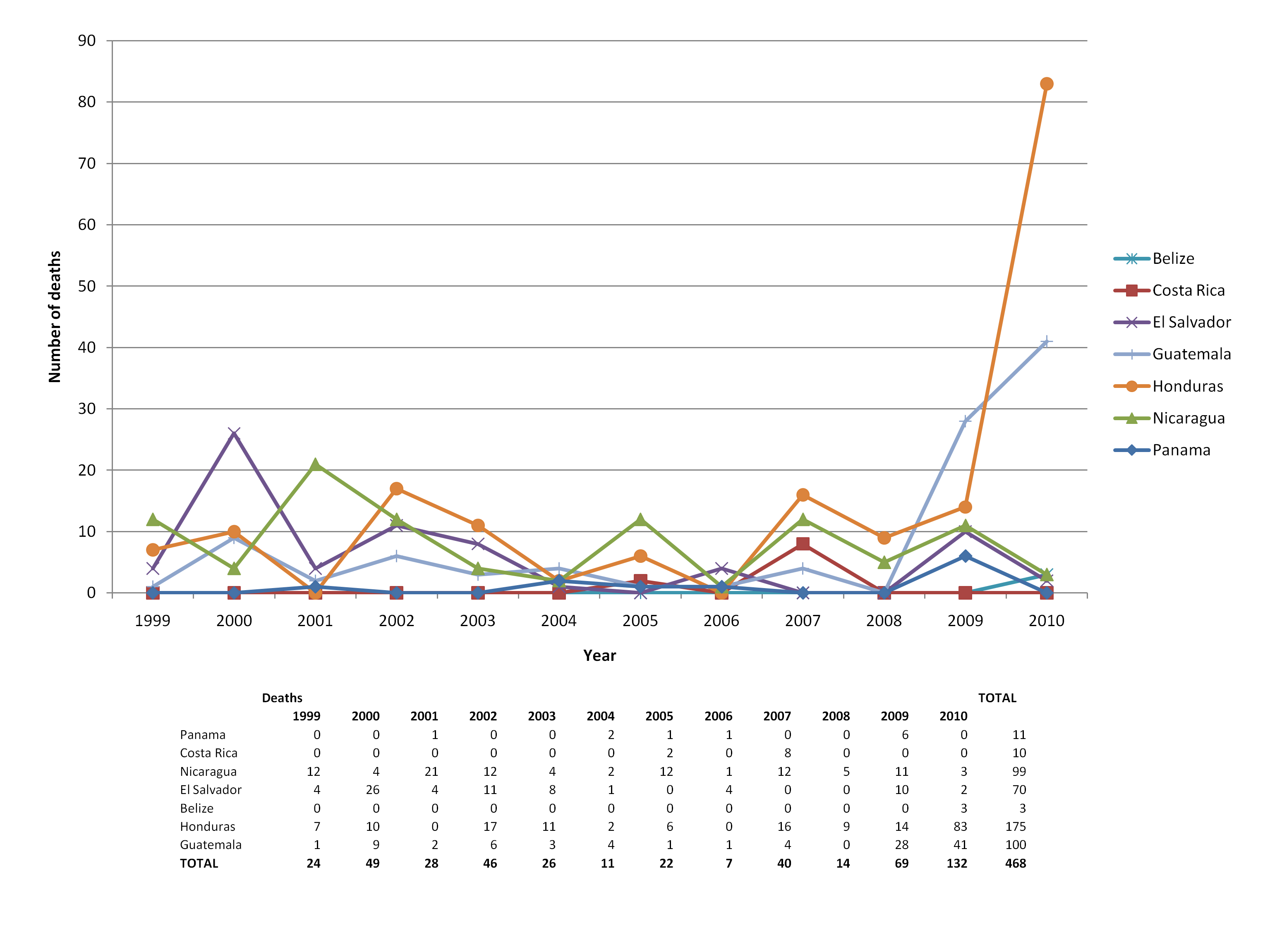

Supplement: Figure S3 — Number of deaths attributed to dengue in countries of Central America from 1999-2010, as reported by the countries to the Pan American Health Organization (PAHO). (TIF) [file pone.0027459.s003.tif]

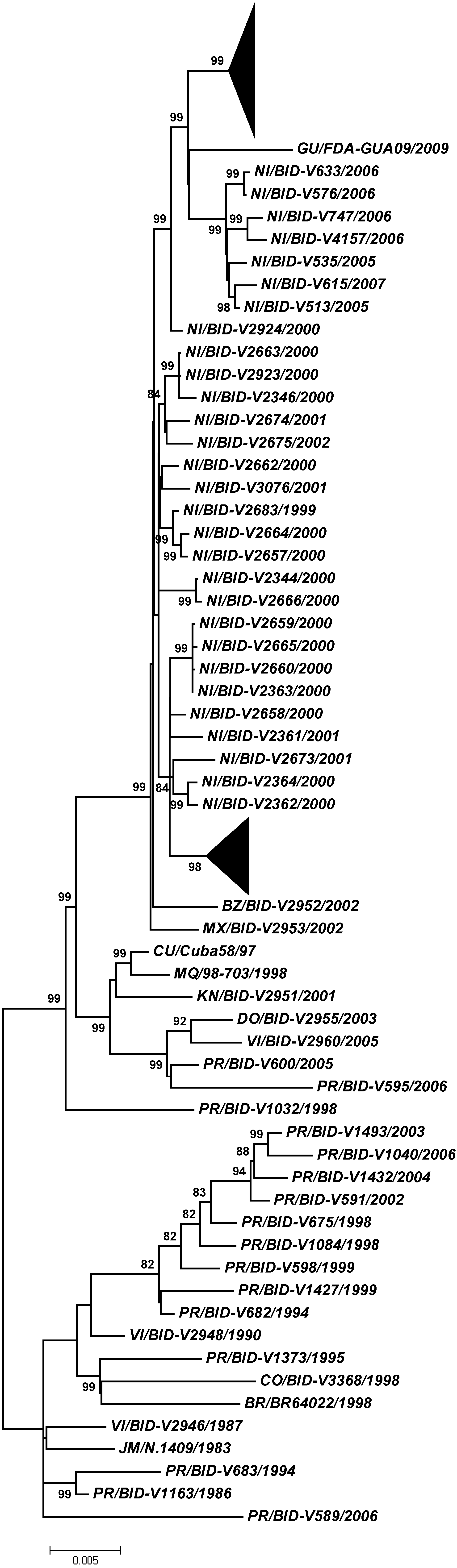

Supplement: Figure S4 — Maximum likelihood consensus tree of the open reading frame of DENV-2 strains from Central America, the Caribbean and South America. (TIF) [file pone.0027459.s004.tif]
