# Supplementary material for: Circulation of Different Lineages of Dengue Virus Type 2 in Central America, Their Evolutionary Time-Scale and Selection Pressure Analysis
Source: PLoS One. 2011 Nov 4;6(11):e27459. doi: 10.1371/journal.pone.0027459 (PMC3208639; doi:10.1371/journal.pone.0027459)
Supplement: Table S1 — List of DENV-2 strains used for phylogenetic, molecular clock and selection pressure analyses, by country and year of isolation. (DOCX) [file pone.0027459.s005.docx]

**Table S1.** List of DENV-2 strains used in this study, country and year of isolation.

| GenBank accession no. | Strain name | Year of Isolation | Country of isolation | Genotype | Phylogenetic analysis | | Selection  analysis | | | | Molecular clock analysis |
| --- | --- | --- | --- | --- | --- | --- | --- | --- | --- | --- | --- |
|  |  |  |  |  | E | ORF | E | | ORF | | E |
|  |  |  |  |  | n=119 | n=158 | n=75 | n=24 | n=143 | n=67 | n=115 |
| M20558 | N.1409 | 1983 | Jamaica | AM/AS | ● |  |  | ● |  |  | ● |
| AF163096 | PTCOL96 | 1996 | Colombia |  | ● |  |  | ● |  |  | ● |
| AF398106 | lard3146 | 1998 | Venezuela |  | ● |  |  | ● |  |  | ● |
| AF208496 | 98-703 | 1998 | Martinique |  | ● | ● | ● | ● | ● | ● | ● |
| FJ898461 | BID-V2952 | 2002 | Belize |  | ● | ● |  |  |  |  |  |
| DQ364515 | ES 18 99 | 1999 | El Salvador |  | ● |  | ● | ● |  |  | ● |
| DQ364514 | CR 7945 00 | 2000 | Costa Rica |  | ● |  | ● | ● |  |  | ● |
| DQ364513 | CR 6530 00 | 2000 | Costa Rica |  | ● |  | ● | ● |  |  | ● |
| AY449682 | 12021/Oxkutzcab | 2001 | Mexico |  | ● |  | ● | ● |  |  | ● |
| AY449683 | 13381/Chochola | 2002 | Mexico |  | ● |  | ● | ● |  |  | ● |
| GU586123 | F07-075 | 2007 | Honduras |  | ● |  | ● | ● |  |  | ● |
| GU586122 | F07-073 | 2007 | Honduras |  | ● |  | ● | ● |  |  | ● |
| GU586492 | F07-030 | 2007 | Guatemala |  | ● |  | ● | ● |  |  | ● |
| HQ999999 | FDA-GUA09 | 2009 | Guatemala |  | ● | ● | ● | ● | ● | ● | ● |
| GQ199895 | BID-V2683 | 1999 | Nicaragua |  | ● | ● | ● | ● | ● | ● | ● |
| FJ850061 | BID-V2346 | 2000 |  |  | ● | ● | ● |  | ● | ● | ● |
| FJ850062 | BID-V2659 | 2000 |  |  | ● | ● | ● |  | ● | ● | ● |
| FJ850065 | BID-V2664 | 2000 |  |  | ● | ● | ● |  | ● | ● | ● |
| FJ744744 | BID-V2364 | 2000 |  |  | ● | ● | ● |  | ● | ● | ● |
| FJ744745 | BID-V2362 | 2000 |  |  | ● | ● | ● |  | ● | ● | ● |
| FJ850117 | BID-V2657 | 2000 |  |  | ● | ● | ● |  | ● | ● | ● |
| FJ850118 | BID-V2658 | 2000 |  |  | ● | ● | ● |  | ● | ● | ● |
| FJ850119 | BID-V2662 | 2000 |  |  | ● | ● | ● |  | ● | ● | ● |
| FJ873808 | BID-V2666 | 2000 |  |  | ● | ● | ● |  | ● | ● | ● |
| FJ898478 | BID-V2924 | 2000 |  |  | ● | ● | ● | ● | ● | ● | ● |
| FJ850120 | BID-V2673 | 2001 |  |  | ● | ● | ● |  | ● | ● | ● |
| FJ850121 | BID-V2674 | 2001 |  |  | ● | ● | ● |  | ● | ● | ● |
| FJ744704 | BID-V2361 | 2001 |  |  | ● | ● | ● |  | ● | ● | ● |
| GQ199898 | BID-V3076 | 2001 |  |  | ● | ● | ● |  | ● | ● | ● |
| GQ199897 | BID-V2675 | 2002 |  |  | ● | ● | ● |  | ● | ● | ● |
| GQ199874 | BID-V668 | 2004 |  |  | ● | ● | ● |  | ● | ● | ● |
| FJ898436 | BID-V627 | 2004 |  |  | ● | ● | ● |  | ● | ● | ● |
| FJ906956 | BID-V543 | 2005 |  |  | ● | ● | ● |  | ● | ● | ● |
| FJ906961 | BID-V2572 | 2005 |  |  | ● | ● | ● |  | ● | ● | ● |
| FJ906962 | BID-V2573 | 2005 |  |  | ● | ● | ● |  | ● | ● | ● |
| HM631868 | BID-V4159 | 2005 |  |  | ● | ● | ● |  | ● | ● | ● |
| HQ541793 | BID-V4636 | 2005 |  |  | ● | ● | ● |  | ● | ● | ● |
| EU482680 | BID-V529 | 2005 |  |  | ● | ● | ● |  | ● | ● | ● |
| EU482695 | BID-V743 | 2005 |  |  | ● | ● | ● |  | ● | ● | ● |
| EU482748 | BID-V513 | 2005 |  |  | ● | ● | ● |  | ● | ● | ● |
| EU482749 | BID-V514 | 2005 |  |  | ● | ● | ● |  | ● | ● | ● |
| EU482750 | BID-V515 | 2005 |  |  | ● | ● | ● |  | ● | ● | ● |
| EU482753 | BID-V524 | 2005 |  |  | ● | ● | ● |  | ● | ● | ● |
| EU482754 | BID-V530 | 2005 |  |  | ● | ● | ● |  | ● | ● | ● |
| EU482755 | BID-V531 | 2005 |  |  | ● | ● | ● |  | ● | ● | ● |
| EU482757 | BID-V535 | 2005 |  |  | ● | ● | ● | ● | ● | ● | ● |
| EU482759 | BID-V544 | 2005 |  |  | ● | ● | ● |  | ● | ● | ● |
| EU482762 | BID-V554 | 2005 |  |  | ● | ● | ● |  | ● | ● | ● |
| EU482763 | BID-V556 | 2005 |  |  | ● | ● | ● |  | ● | ● | ● |
| EU482766 | BID-V693 | 2005 |  |  | ● | ● |  |  | ● |  | ● |
| FJ478455 | BID-V532 | 2005 |  |  | ● | ● |  |  | ● |  | ● |
| FJ850115 | BID-V634 | 2005 |  |  | ● | ● | ● |  | ● | ● | ● |
| FJ850067 | BID-V2331 | 2006 |  |  | ● | ● | ● |  | ● | ● | ● |
| EU482444 | BID-V563 | 2006 |  |  | ● | ● | ● |  | ● | ● | ● |
| EU482634 | BID-V633 | 2006 |  |  | ● | ● | ● | ● | ● | ● | ● |
| EU482685 | BID-V572 | 2006 |  |  | ● | ● |  |  | ● |  | ● |
| EU482687 | BID-V574 | 2006 |  |  | ● | ● | ● |  | ● | ● | ● |
| EU482688 | BID-V575 | 2006 |  |  | ● | ● | ● |  | ● | ● | ● |
| EU482689 | BID-V576 | 2006 |  |  | ● | ● | ● |  | ● | ● | ● |
| EU482692 | BID-V580 | 2006 |  |  | ● | ● |  |  | ● |  | ● |
| EU482696 | BID-V747 | 2006 |  |  | ● | ● | ● | ● | ● | ● | ● |
| FJ744741 | BID-V1762 | 2006 |  |  | ● | ● | ● |  | ● | ● | ● |
| FJ744742 | BID-V1763 | 2006 |  |  | ● | ● |  |  | ● |  | ● |
| HM631866 | BID-V4157 | 2006 |  |  | ● | ● | ● |  | ● | ● | ● |
| HM631867 | BID-V4158 | 2006 |  |  | ● | ● | ● |  | ● | ● | ● |
| HQ541786 | BID-V2596 | 2006 |  |  | ● | ● |  |  | ● |  | ● |
| HQ541787 | BID-V3136 | 2007 |  |  | ● | ● |  |  | ● |  | ● |
| EU482603 | BID-V615 | 2007 |  |  | ● | ● | ● |  | ● | ● | ● |
| EU482623 | BID-V1210 | 2007 |  |  | ● | ● | ● |  | ● | ● | ● |
| EU482624 | BID-V1229 | 2007 |  |  | ● | ● | ● |  | ● | ● | ● |
| EU482625 | BID-V1232 | 2007 |  |  | ● | ● | ● |  | ● | ● | ● |
| EU482627 | BID-V1235 | 2007 |  |  | ● | ● | ● |  | ● | ● | ● |
| EU482629 | BID-V1297 | 2007 |  |  | ● | ● | ● |  | ● | ● | ● |
| EU569702 | BID-V1228 | 2007 |  |  | ● | ● | ● |  | ● | ● | ● |
| EU596497 | BID-V1202 | 2007 |  |  | ● | ● |  |  | ● |  | ● |
| FJ850050 | BID-V2356 | 2007 |  |  | ● | ● |  |  | ● |  | ● |
| FJ850051 | BID-V2635 | 2007 |  |  | ● | ● | ● |  | ● | ● | ● |
| FJ547090 | BID-V1311 | 2007 |  |  | ● | ● | ● |  | ● | ● | ● |
| FJ639833 | BID-V1237 | 2007 |  |  | ● | ● |  |  | ● |  | ● |
| FJ639834 | BID-V2354 | 2007 |  |  | ● | ● |  |  | ● |  | ● |
| FJ639837 | BID-V2358 | 2007 |  |  | ● | ● | ● | ● | ● | ● | ● |
| FJ744703 | BID-V1764 | 2007 |  |  | ● | ● | ● |  | ● | ● | ● |
| FJ744706 | BID-V2359 | 2007 |  |  | ● | ● | ● |  | ● | ● | ● |
| FJ744707 | BID-V2360 | 2007 |  |  | ● | ● | ● |  | ● | ● | ● |
| FJ744743 | BID-V1755 | 2007 |  |  | ● | ● |  |  | ● |  | ● |
| FJ882594 | BID-V1313 | 2007 |  |  | ● | ● | ● |  | ● | ● | ● |
| FJ898432 | BID-V1304 | 2007 |  |  | ● | ● |  |  | ● |  | ● |
| GQ868646 | BID-V2428 | 2007 |  |  | ● | ● | ● |  | ● | ● | ● |
| FJ205885 | BID-V1721 | 2008 |  |  | ● | ● | ● |  | ● | ● | ● |
| FJ810418 | BID-V2353 | 2008 |  |  | ● | ● | ● |  | ● | ● | ● |
| HQ705624 | BID-V4914 | 2009 |  |  | ● | ● | ● |  | ● | ● | ● |
| HQ705625 | BID-V4915 | 2009 |  |  | ● | ● | ● |  | ● | ● | ● |
| FJ744705 | BID-V2363 | 2000 |  |  |  | ● |  |  | ● |  |  |
| FJ850060 | BID-V2344 | 2000 |  |  |  | ● |  |  | ● |  |  |
| FJ850063 | BID-V2660 | 2000 |  |  |  | ● |  |  | ● |  |  |
| FJ850064 | BID-V2663 | 2000 |  |  |  | ● |  |  | ● |  |  |
| FJ850066 | BID-V2665 | 2000 |  |  |  | ● |  |  | ● |  |  |
| FJ898477 | BID-V2923 | 2000 |  |  |  | ● |  |  | ● |  |  |
| EU482597 | BID-V609 | 2005 |  |  |  | ● |  |  | ● |  |  |
| EU482635 | BID-V640 | 2005 |  |  |  | ● |  |  | ● |  |  |
| EU482751 | BID-V517 | 2005 |  |  |  | ● |  |  | ● |  |  |
| EU482760 | BID-V548 | 2005 |  |  |  | ● |  |  | ● |  |  |
| EU482761 | BID-V553 | 2005 |  |  |  | ● |  |  | ● |  |  |
| EU482769 | BID-V744 | 2005 |  |  |  | ● |  |  | ● |  |  |
| EU482770 | BID-V527 | 2005 |  |  |  | ● |  |  | ● |  |  |
| FJ226066 | BID-V528 | 2005 |  |  |  | ● |  |  | ● |  |  |
| FJ850053 | BID-V2574 | 2005 |  |  |  | ● |  |  | ● |  |  |
| FJ850054 | BID-V2576 | 2005 |  |  |  | ● |  |  | ● |  |  |
| GQ868604 | BID-V518 | 2005 |  |  |  | ● |  |  | ● |  |  |
| HQ541794 | BID-V4639 | 2005 |  |  |  | ● |  |  | ● |  |  |
| EU482620 | BID-V1074 | 2006 |  |  |  | ● |  |  | ● |  |  |
| EU482639 | BID-V673 | 2006 |  |  |  | ● |  | ● | ● |  |  |
| EU482682 | BID-V559 | 2006 |  |  |  | ● |  |  | ● |  |  |
| EU482686 | BID-V573 | 2006 |  |  |  | ● |  |  | ● |  |  |
| EU482691 | BID-V579 | 2006 |  |  |  | ● |  |  | ● |  |  |
| EU482772 | BID-V565 | 2006 |  |  |  | ● |  |  | ● |  |  |
| EU596483 | BID-V608 | 2006 |  |  |  | ● |  | ● | ● |  |  |
| GQ199896 | BID-V2680 | 2006 |  |  |  | ● |  |  | ● |  |  |
| HQ733861 | BID-V2599 | 2006 |  |  |  | ● |  |  | ● |  |  |
| EU482622 | BID-V1198 | 2007 |  |  |  | ● |  |  | ● |  |  |
| EU482630 | BID-V1312 | 2007 |  |  |  | ● |  |  | ● |  |  |
| EU482638 | BID-V662 | 2007 |  |  |  | ● |  |  | ● |  |  |
| EU482694 | BID-V582 | 2007 |  |  |  | ● |  |  | ● |  |  |
| EU569692 | BID-V1192 | 2007 |  |  |  | ● |  |  | ● |  |  |
| EU569693 | BID-V1193 | 2007 |  |  |  | ● |  |  | ● |  |  |
| EU569695 | BID-V1203 | 2007 |  |  |  | ● |  |  | ● |  |  |
| EU569697 | BID-V1208 | 2007 |  |  |  | ● |  |  | ● |  |  |
| EU569698 | BID-V1212 | 2007 |  |  |  | ● |  |  | ● |  |  |
| EU569699 | BID-V1217 | 2007 |  |  |  | ● |  |  | ● |  |  |
| EU569700 | BID-V1219 | 2007 |  |  |  | ● |  |  | ● |  |  |
| EU569701 | BID-V1224 | 2007 |  |  |  | ● |  |  | ● |  |  |
| EU596495 | BID-V1195 | 2007 |  |  |  | ● |  |  | ● |  |  |
| EU596496 | BID-V1200 | 2007 |  |  |  | ● |  |  | ● |  |  |
| EU596498 | BID-V1211 | 2007 |  |  |  | ● |  |  | ● |  |  |
| EU596499 | BID-V1215 | 2007 |  |  |  | ● |  |  | ● |  |  |
| EU596500 | BID-V1230 | 2007 |  |  |  | ● |  |  | ● |  |  |
| EU660404 | BID-V1196 | 2007 |  |  |  | ● |  |  | ● |  |  |
| EU660405 | BID-V1201 | 2007 |  |  |  | ● |  |  | ● |  |  |
| FJ373300 | BID-V1194 | 2007 |  |  |  | ● |  |  | ● |  |  |
| FJ390390 | BID-V1298 | 2007 |  |  |  | ● |  |  | ● |  |  |
| FJ390391 | BID-V1300 | 2007 |  |  |  | ● |  |  | ● |  |  |
| FJ410291 | BID-V1233 | 2007 |  |  |  | ● |  |  | ● |  |  |
| FJ478459 | BID-V1214 | 2007 |  |  |  | ● |  |  | ● |  |  |
| FJ639835 | BID-V2355 | 2007 |  |  |  | ● |  |  | ● |  |  |
| FJ639836 | BID-V2357 | 2007 |  |  |  | ● |  |  | ● |  |  |
| FJ744708 | BID-V2352 | 2007 |  |  |  | ● |  |  | ● |  |  |
| FJ850116 | BID-V1197 | 2007 |  |  |  | ● |  |  | ● |  |  |
| FJ882593 | BID-V1302 | 2007 |  |  |  | ● |  |  | ● |  |  |
| GQ199869 | BID-V3002 | 2007 |  |  |  | ● |  | ● | ● |  |  |
| HQ541788 | BID-V3149 | 2007 |  |  |  | ● |  |  | ● |  |  |
| JF357905 | BID-V2605 | 2007 |  |  |  | ● |  |  | ● |  |  |
| EU482621 | BID-V1191 | 2007 |  |  |  |  |  | ● |  |  |  |
| FJ906960 | BID-V2569 | 2007 |  |  |  |  |  | ● |  |  |  |
| FJ744709 | BID-V2351 | 2008 |  |  |  | ● |  |  | ● |  |  |
| JF357906 | BID-V3227 | 2008 |  |  |  | ● |  |  | ● |  |  |
| JF730051 | BID-V5072 | 2009 |  |  |  | ● |  |  | ● |  |  |
| JF730052 | BID-V5073 | 2009 |  |  |  | ● |  |  | ● |  |  |
| L10053 | TRIN-53 | 1953 | Trinidad | AM | ● |  |  |  |  |  | ● |
| L10043 | India | 1957 | India |  | ● |  |  |  |  |  | ● |
| L10046 | PR159 | 1969 | Puerto Rico |  | ● |  |  |  |  |  | ● |
| AY744147 | Tonga74 | 1974 | Tonga |  | ● | ● |  |  |  |  | ● |
| AF100458 | I348600 | 1986 | Colombia |  | ● | ● |  |  |  |  | ● |
| AF100465 | Ven2 | 1987 | Venezuela |  | ● | ● |  |  |  |  | ● |
| AF100469 | 0131 | 1992 | Mexico |  | ● | ● |  |  |  |  | ● |
| AF100468 | IQT2913 | 1996 | Peru |  | ● | ● |  |  |  |  | ● |
| D10514 | TH-36 | 1958 | Thailand | AS-I | ● |  |  |  |  |  | ● |
| DQ181805 | ThD2 0168 | 1979 | Thailand |  | ● | ● |  |  |  |  | ● |
| DQ181804 | ThD2 0498 | 1984 | Thailand |  | ● | ● |  |  |  |  | ● |
| X15434 | M1 | 1987 | Malaysia |  | ● |  |  |  |  |  | ● |
| D00345 | PUO-218 | 1989 | Thailand |  | ● |  |  |  |  |  | ● |
| DQ181797 | ThD2 0078 | 2001 | Thailand |  | ● | ● |  |  |  |  | ● |
| AF038403 | New Guinea C | 1944 | New Guinea | AS-II | ● | ● |  |  |  |  | ● |
| L10045 | PHILIP | 1983 | Philippines |  | ● |  |  |  |  |  | ● |
| L10052 | Taiwan | 1987 | Taiwan |  | ● |  |  |  |  |  | ● |
| L10044 | Indon | 1976 | Indonesia | COS | ● |  |  |  |  |  | ● |
| L10051 | Somalia | 1984 | Somalia |  | ● |  |  |  |  |  | ● |
| AF410370 | CAMR5 | 1992 | Australia |  | ● |  |  |  |  |  | ● |
| AY037116 | TSV01 | 1993 | Australia |  | ● | ● |  |  |  |  | ● |
| AB189122 | 98900663 | 1998 | Indonesia |  | ● | ● |  |  |  |  | ● |
| AF359579 | FJ11 | 1999 | China |  | ● | ● |  |  |  |  | ● |
| AY858035 | BA05i | 2004 | Indonesia |  | ● | ● |  |  |  |  | ● |
| AF231719 | PM33974 | 1981 | Guinea | Sylvatic | ● | ● |  |  |  |  |  |
| AF231717 | P8-1407 | 1970 | Malaysia |  | ● |  |  |  |  |  |  |
| AF231718 | DAKAr578 | 1980 | Ivory Coast |  | ● |  |  |  |  |  |  |

(●) denotes that the strain was included in the described analysis; AM, American; ASI, Asian I; ASII, Asian II; CO, Cosmopolitan; AM/AS, American/Asian genotypes. E, envelope; ORF, open reading frame.
