# Supplementary material for: Circulation of Different Lineages of Dengue Virus Type 2 in Central America, Their Evolutionary Time-Scale and Selection Pressure Analysis
Source: PLoS One. 2011 Nov 4;6(11):e27459. doi: 10.1371/journal.pone.0027459 (PMC3208639; doi:10.1371/journal.pone.0027459)
Supplement: Table S2 — List of DENV-2 strains of American/Asian genotype used for phylogenetic analysis of ORF sequences between circulating strains of Central American and Caribbean origin (n = 170), by country, year of isolation and clade. (DOCX) [file pone.0027459.s006.docx]

**Table S2.** List of DENV-2 strains of American/Asian genotype used for phylogenetic analysis of ORF sequences between circulating strains of Central American and Caribbean origin (n=170), by country, year of isolation and clade.

| GenBank accession no. | Strain name | Year of Isolation | Country of isolation | Clade |
| --- | --- | --- | --- | --- |
| AF489932 | BR64022 | 1998 | Brazil | 1 |
| GQ868552 | BID-V3368 | 1998 | Colombia | 1 |
| AY702035 | Cuba58 | 1997 | Cuba | 2 |
| FJ898451 | BID-2955 | 2003 | Dominican Republic | 2 |
| M20558 | N.1409 | 1983 | Jamaica | 1 |
| AF208496 | 98-703 | 1998 | Martinique | 2 |
| FJ898438 | BID-V2953 | 2002 | Mexico | 2a |
| EU569704 | BID-V1163 | 1986 | Puerto Rico | 1 |
| EU482739 | BID-V682 | 1994 |  | 1 |
| EU482740 | BID-V683 | 1994 |  | 1 |
| EU569708 | BID-V1373 | 1995 |  | 1 |
| EU482545 | BID-V1032 | 1998 |  | 2 |
| EU482565 | BID-V1084 | 1998 |  | 1 |
| EU482732 | BID-V675 | 1998 |  | 1 |
| EU482729 | BID-V598 | 1999 |  | 1 |
| EU677144 | BID-V1427 | 1999 |  | 1 |
| EU482722 | BID-V591 | 2002 |  | 1 |
| EU687241 | BID-V1493 | 2003 |  | 1 |
| EU677147 | BID-V1432 | 2004 |  | 1 |
| EU482731 | BID-V600 | 2005 |  | 2 |
| EU482552 | BID-V1040 | 2006 |  | 1 |
| EU482721 | BID-V589 | 2006 |  | 1 |
| EU482726 | BID-V595 | 2006 |  | 2 |
| FJ898460 | BID-V2951 | 2001 | St. Kitts and Nevis | 2 |
| GQ868603 | BID-V2946 | 1987 | US Virgin Islands | 1 |
| FJ898450 | BID-V2948 | 1990 | US Virgin Islands | 1 |
| FJ898453 | BID-V2960 | 2005 | US Virgin Islands | 2 |
| FJ898461 | BID-V2952 | 2002 | Belize | 2a |
| HQ999999 | FDA-GUA09 | 2009 | Guatemala | 2b |
| GQ199895 | BID-V2683 | 1999 | Nicaragua | 2a |
| FJ850061 | BID-V2346 | 2000 |  | 2a |
| FJ850062 | BID-V2659 | 2000 |  | 2a |
| FJ850065 | BID-V2664 | 2000 |  | 2a |
| FJ744744 | BID-V2364 | 2000 |  | 2a |
| FJ744745 | BID-V2362 | 2000 |  | 2a |
| FJ850117 | BID-V2657 | 2000 |  | 2a |
| FJ850118 | BID-V2658 | 2000 |  | 2a |
| FJ850119 | BID-V2662 | 2000 |  | 2a |
| FJ873808 | BID-V2666 | 2000 |  | 2a |
| FJ898478 | BID-V2924 | 2000 |  | 2b |
| FJ850120 | BID-V2673 | 2001 |  | 2a |
| FJ850121 | BID-V2674 | 2001 |  | 2a |
| FJ744704 | BID-V2361 | 2001 |  | 2a |
| GQ199898 | BID-V3076 | 2001 |  | 2a |
| GQ199897 | BID-V2675 | 2002 |  | 2a |
| GQ199874 | BID-V668 | 2004 |  | 2a |
| FJ898436 | BID-V627 | 2004 |  | 2a |
| FJ906956 | BID-V543 | 2005 |  | 2a |
| FJ906961 | BID-V2572 | 2005 |  | 2a |
| FJ906962 | BID-V2573 | 2005 |  | 2a |
| HM631868 | BID-V4159 | 2005 |  | 2a |
| HQ541793 | BID-V4636 | 2005 |  | 2a |
| EU482680 | BID-V529 | 2005 |  | 2a |
| EU482695 | BID-V743 | 2005 |  | 2a |
| EU482748 | BID-V513 | 2005 |  | 2b |
| EU482749 | BID-V514 | 2005 |  | 2a |
| EU482750 | BID-V515 | 2005 |  | 2a |
| EU482753 | BID-V524 | 2005 |  | 2a |
| EU482754 | BID-V530 | 2005 |  | 2a |
| EU482755 | BID-V531 | 2005 |  | 2a |
| EU482757 | BID-V535 | 2005 |  | 2b |
| EU482759 | BID-V544 | 2005 |  | 2a |
| EU482762 | BID-V554 | 2005 |  | 2a |
| EU482763 | BID-V556 | 2005 |  | 2a |
| EU482766 | BID-V693 | 2005 |  | 2b |
| FJ478455 | BID-V532 | 2005 |  | 2b |
| FJ850115 | BID-V634 | 2005 |  | 2a |
| FJ850067 | BID-V2331 | 2006 |  | 2a |
| EU482444 | BID-V563 | 2006 |  | 2a |
| EU482634 | BID-V633 | 2006 |  | 2b |
| EU482685 | BID-V572 | 2006 |  | 2b |
| EU482687 | BID-V574 | 2006 |  | 2b |
| EU482688 | BID-V575 | 2006 |  | 2a |
| EU482689 | BID-V576 | 2006 |  | 2b |
| EU482692 | BID-V580 | 2006 |  | 2b |
| EU482696 | BID-V747 | 2006 |  | 2b |
| FJ744741 | BID-V1762 | 2006 |  | 2a |
| FJ744742 | BID-V1763 | 2006 |  | 2b |
| HM631866 | BID-V4157 | 2006 |  | 2b |
| HM631867 | BID-V4158 | 2006 |  | 2b |
| HQ541786 | BID-V2596 | 2006 |  | 2b |
| HQ541787 | BID-V3136 | 2007 |  | 2b |
| EU482603 | BID-V615 | 2007 |  | 2b |
| EU482623 | BID-V1210 | 2007 |  | 2b |
| EU482624 | BID-V1229 | 2007 |  | 2b |
| EU482625 | BID-V1232 | 2007 |  | 2b |
| EU482627 | BID-V1235 | 2007 |  | 2b |
| EU482629 | BID-V1297 | 2007 |  | 2a |
| EU569702 | BID-V1228 | 2007 |  | 2b |
| EU596497 | BID-V1202 | 2007 |  | 2b |
| FJ850050 | BID-V2356 | 2007 |  | 2b |
| FJ850051 | BID-V2635 | 2007 |  | 2b |
| FJ547090 | BID-V1311 | 2007 |  | 2b |
| FJ639833 | BID-V1237 | 2007 |  | 2b |
| FJ639834 | BID-V2354 | 2007 |  | 2b |
| FJ639837 | BID-V2358 | 2007 |  | 2b |
| FJ744703 | BID-V1764 | 2007 |  | 2b |
| FJ744706 | BID-V2359 | 2007 |  | 2b |
| FJ744707 | BID-V2360 | 2007 |  | 2b |
| FJ744743 | BID-V1755 | 2007 |  | 2b |
| FJ882594 | BID-V1313 | 2007 |  | 2a |
| FJ898432 | BID-V1304 | 2007 |  | 2b |
| GQ868646 | BID-V2428 | 2007 |  | 2a |
| FJ205885 | BID-V1721 | 2008 |  | 2b |
| FJ810418 | BID-V2353 | 2008 |  | 2b |
| HQ705624 | BID-V4914 | 2009 |  | 2a |
| HQ705625 | BID-V4915 | 2009 |  | 2b |
| FJ744705 | BID-V2363 | 2000 |  | 2a |
| FJ850060 | BID-V2344 | 2000 |  | 2a |
| FJ850063 | BID-V2660 | 2000 |  | 2a |
| FJ850064 | BID-V2663 | 2000 |  | 2a |
| FJ850066 | BID-V2665 | 2000 |  | 2a |
| FJ898477 | BID-V2923 | 2000 |  | 2a |
| EU482597 | BID-V609 | 2005 |  | 2a |
| EU482635 | BID-V640 | 2005 |  | 2a |
| EU482751 | BID-V517 | 2005 |  | 2b |
| EU482760 | BID-V548 | 2005 |  | 2a |
| EU482761 | BID-V553 | 2005 |  | 2a |
| EU482769 | BID-V744 | 2005 |  | 2a |
| EU482770 | BID-V527 | 2005 |  | 2a |
| FJ226066 | BID-V528 | 2005 |  | 2a |
| FJ850053 | BID-V2574 | 2005 |  | 2a |
| FJ850054 | BID-V2576 | 2005 |  | 2a |
| GQ868604 | BID-V518 | 2005 |  | 2b |
| HQ541793 | BID-V4636 | 2005 |  | 2a |
| HQ541794 | BID-V4639 | 2005 |  | 2a |
| EU482620 | BID-V1074 | 2006 |  | 2b |
| EU482639 | BID-V673 | 2006 |  | 2a |
| EU482682 | BID-V559 | 2006 |  | 2b |
| EU482686 | BID-V573 | 2006 |  | 2b |
| EU482691 | BID-V579 | 2006 |  | 2b |
| EU482772 | BID-V565 | 2006 |  | 2b |
| EU596483 | BID-V608 | 2006 |  | 2b |
| GQ199896 | BID-V2680 | 2006 |  | 2b |
| HQ733861 | BID-V2599 | 2006 |  | 2b |
| EU482622 | BID-V1198 | 2007 |  | 2b |
| EU482630 | BID-V1312 | 2007 |  | 2b |
| EU482638 | BID-V662 | 2007 |  | 2b |
| EU482694 | BID-V582 | 2007 |  | 2b |
| EU569692 | BID-V1192 | 2007 |  | 2b |
| EU569693 | BID-V1193 | 2007 |  | 2b |
| EU569695 | BID-V1203 | 2007 |  | 2b |
| EU569697 | BID-V1208 | 2007 |  | 2b |
| EU569698 | BID-V1212 | 2007 |  | 2b |
| EU569699 | BID-V1217 | 2007 |  | 2a |
| EU569700 | BID-V1219 | 2007 |  | 2b |
| EU569701 | BID-V1224 | 2007 |  | 2b |
| EU596495 | BID-V1195 | 2007 |  | 2b |
| EU596496 | BID-V1200 | 2007 |  | 2b |
| EU596498 | BID-V1211 | 2007 |  | 2b |
| EU596499 | BID-V1215 | 2007 |  | 2b |
| EU596500 | BID-V1230 | 2007 |  | 2b |
| EU660404 | BID-V1196 | 2007 |  | 2b |
| EU660405 | BID-V1201 | 2007 |  | 2b |
| FJ373300 | BID-V1194 | 2007 |  | 2b |
| FJ390390 | BID-V1298 | 2007 |  | 2a |
| FJ390391 | BID-V1300 | 2007 |  | 2b |
| FJ410291 | BID-V1233 | 2007 |  | 2a |
| FJ478459 | BID-V1214 | 2007 |  | 2b |
| FJ639835 | BID-V2355 | 2007 |  | 2b |
| FJ639836 | BID-V2357 | 2007 |  | 2b |
| FJ744708 | BID-V2352 | 2007 |  | 2b |
| FJ850116 | BID-V1197 | 2007 |  | 2b |
| FJ882593 | BID-V1302 | 2007 |  | 2b |
| GQ199869 | BID-V3002 | 2007 |  | 2b |
| HQ541788 | BID-V3149 | 2007 |  | 2a |
| JF357905 | BID-V2605 | 2007 |  | 2b |
| FJ744709 | BID-V2351 | 2008 |  | 2b |
| JF357906 | BID-V3227 | 2008 |  | 2b |
| JF730051 | BID-V5072 | 2009 |  | 2b |
| JF730052 | BID-V5073 | 2009 |  | 2b |

Number of strains by country: Nicaragua = 141, Guatemala = 1, Belize = 1, Mexico = 1, Puerto Rico = 16
US Virgin Islands = 3, Saint Kitts and Nevis = 1, Martinique = 1, Jamaica = 1, Dominican Republic = 1, Cuba = 1, Colombia = 1, Brazil = 1
