# Supplementary material for: Circulation of Different Lineages of Dengue Virus Type 2 in Central America, Their Evolutionary Time-Scale and Selection Pressure Analysis
Source: PLoS One. 2011 Nov 4;6(11):e27459. doi: 10.1371/journal.pone.0027459 (PMC3208639; doi:10.1371/journal.pone.0027459)
Supplement: Table S3 — Molecular clock analysis of DENV-2 from all genotypes used in the study, showing the Effective Sample Size (ESS) for the studied parameters. (DOCX) [file pone.0027459.s007.docx]

**Table S3.** Molecular clock analysis of DENV-2 from all genotypes used in the study, showing the Effective Sample Size (ESS) for the studied parameters.

Best-fit model is shown in bold. GTR + Γ_4_ + I, General Time Reversible (GTR) substitution model with 4 categories of Γ plus invariable rates; 95% HPD, 95% highest probability density; TMRCA, time to the most recent common ancestor.

| Model | Prior (mean) | Posterior (mean) | Marginal likelihood (mean ± stderr) | Substitution rate mean  (10^–4^ substitutions/site/year [95%HPD]) | TMRCA (Root age)  (mean no. of years [95%HPD]) |
| --- | --- | --- | --- | --- | --- |
| Bayesian coalescent prior: |  |  |  |  |  |
| **GTR + Γ + I, relaxed lognormal clock** | **-538.11** | **-8018.52** | **-7480.41 ± 0.34** | **7.46**  **(6.32–8.68)** | **120.61**  **(105.14–137.97)** |
| ESS | 340.02 | 427.164 | 961.58 | 1650.83 | 2177.96 |
|  |  |  |  |  |  |
| GTR + Γ + I, strict clock | -548.27 | -8030.81 | -7482.53 ± 0.35 | 7.58  (6.43–8.79) | 118.88  (103.93–135.03) |
| ESS | 1672.90 | 1002.79 | 922.43 | 2544.13 | 4535.78 |
| Constant size: |  |  |  |  |  |
| GTR + Γ + I, relaxed lognormal clock | -538.73 | -8022.66 | -7483.93 ± 0.33 | 7.49  (6.38–8.63) | 119.99  (106.40–134.62) |
| ESS | 657.45 | 704.39 | 893.30 | 2447.27 | 3954.56 |
|  |  |  |  |  |  |
| GTR + Γ + I, strict clock | -549.27 | -8035.01 | -7485.73 ± 0.24 | 7.59  (6.48–8.80) | 118.39  (105.38–132.73) |
| ESS | 2768.95 | 1658.71 | 1814.31 | 4316.47 | 7202.24 |
